# Whole-body magnetic resonance imaging in pediatric oncology — recommendations by the Oncology Task Force of the ESPR

**DOI:** 10.1007/s00247-020-04683-4

**Published:** 2020-05-28

**Authors:** Jürgen F. Schäfer, Claudio Granata, Thekla von Kalle, Martin Kyncl, Annemieke S. Littooij, Pier Luigi Di Paolo, Irmina Sefic Pasic, Rutger A. J. Nievelstein, on behalf of the Oncology Task Force of the ESPR

**Affiliations:** 1 Division of Pediatric Radiology, Department of Radiology, University Hospital of Tübingen, Hoppe-Seyler-Straße 3, 72076 Tübingen, Germany; 2 Department of Paediatric Radiology, IRCCS materno infantile Burlo Garofolo, Trieste, Italy; 3 Department of Pediatric Radiology, Olgahospital Klinikum Stuttgart, Stuttgart, Germany; 4 Department of Pediatric Radiology, Charles University and Motol University Hospital, Prague, Czech Republic; 5 Department of Radiology & Nuclear Medicine, Princess Maxima Center for Pediatric Oncology, Wilhelmina Children’s Hospital/University Medical Center Utrecht, Utrecht, The Netherlands; 6 Radiology Department, Bambino Gesù Children’s Hospital, Rome, Italy; 7 Radiology Clinic, Sarajevo School of Science and Technology, Clinical Center University of Sarajevo, Sarajevo, Bosnia and Herzegovina

**Keywords:** Cancer predisposition syndromes, Children, Magnetic resonance imaging, Neoplasia, Staging, Surveillance, Whole-body imaging

## Abstract

The purpose of this recommendation of the Oncology Task Force of the European Society of Paediatric Radiology (ESPR) is to indicate reasonable applications of whole-body MRI in children with cancer and to address useful protocols to optimize workflow and diagnostic performance. Whole-body MRI as a radiation-free modality has been increasingly performed over the last two decades, and newer applications, as in screening of children with germ-line mutation cancer-related gene defects, are now widely accepted. We aim to provide a comprehensive outline of the diagnostic value for use in daily practice. Based on the results of our task force session in 2018 and the revision in 2019 during the ESPR meeting, we summarized our group’s experiences in whole-body MRI. The lack of large evidence by clinical studies is challenging when focusing on a balanced view regarding the impact of whole-body MRI in pediatric oncology. Therefore, the final version of this recommendation was supported by the members of Oncology Task Force.

## Introduction

Whole-body MRI has been increasingly used in diagnosing pediatric oncological and non-oncological diseases over the last two decades [1–13]. Whole-body MRI is defined as a radiation-free image modality that allows a comprehensive investigation with a maximal scan length from head to toe, or minimal from base of skull to upper thigh, within a reasonable time frame. It comprises the simultaneous imaging of bone, bone marrow, soft tissue and the central nervous system. There are two ways of using this method: on the one hand, whole-body MRI is performed as a screening tool in addition to the standard imaging, mostly in coronal plane with short tau inversion recovery (STIR) sequences alone or in combination with T1-weighted images [1, 14, 15]. The advantage of short examination time has to be balanced with lower diagnostic performance in comparison to local MRI examinations. On the other hand, using both modern scanners as well as advanced sequences, the spatial resolution and the specific MRI contrasts are similar to those in local examination settings, with only some limitation regarding image quality of the extremities [13, 16–18]. In addition, the possibility of combining local and whole-body imaging represents a distinct advantage of the method, also allowing functional imaging (e.g., diffusion or perfusion methods) [9, 19–23]. However, to keep the examination time in an acceptable range, particularly regarding patient comfort and safety, intelligent and dedicated sequence protocols are recommended. Moreover, the high sensitivity of MRI regarding bone marrow changes has to be considered to avoid overinterpretation of normal findings in children [24]. Therefore, for specific applications and diagnostic confidence, an optimized agreement of sequence numbers, spatial resolution and acquisition time must be realized. This issue is unquestionably challenging and needs further harmonization.

Regarding local staging and response assessment of solid malignant tumors in children and adolescents, MRI is the modality of choice in most cases, whereas whole-body MRI represents a non-standard method and is not generally considered in the protocols of the international oncological pediatric societies (e.g., International Society of Paediatric Oncology, or SIOP). However, in contrast, at a number of institutions whole-body MRI is now integrated into the diagnostic workup in children with cancer. Unfortunately, the diagnostic value has been evaluated mostly in small single-center projects. It is primarily in this context that the value of whole-body MRI for detecting osteomedullary metastases has been assessed [1, 3, 15, 25, 26]. But tumor spread into the abdominal organs, lymph nodes and the central nervous system has also been increasingly investigated with whole-body MRI [2, 6, 7, 27–29], with increasing accuracy when diffusion-weighted sequences are added to the imaging protocol [9, 30]. Most trials have compared the diagnostic accuracy of whole-body MRI with conventionally used imaging workups including metabolic imaging. Only a few studies have measured the predictive value of whole-body MRI findings or the change in patient outcome [5]. A relatively new application of whole-body MRI is its use in cancer predisposition disorders as a significant screening procedure, which is now established for some diseases, with an increasing database (e.g., Li–Fraumeni syndrome) [11, 31–38].

In summary, on the one hand, whole-body MRI exists as a valuable tool in the daily practice of pediatric oncology and is frequently performed in specialized centers. On the other hand, there are major concerns about the level of scientific evidence. Hence, the presented recommendations summarize a consensus statement by experts in the field of whole-body MRI where large evidence is lacking. Finally, the confusion about the protocols and techniques needs to be harmonized. Therefore, this recommendation of the Oncology Task Force aims to specify applications of whole-body MRI, to describe the technical pre-requisition, and to define useful protocols to maximize the diagnostic accuracy and confidence.

## Key recommendations

- Whole-body MRI enables the investigation of systemic spread of solid malignant tumors.
- Whole-body MRI is useful for surveillance in cancer predisposition syndromes.
- Imaging protocol adaption to each child’s age and the indication is mandatory to maximize diagnostic performance.
- The high sensitivity of STIR MRI regarding bone marrow changes has to be considered to avoid overinterpretation of normal findings.
- Additional imaging might be necessary in particular tumor entities or diagnostic scenarios.

## Specific tumors

### Lymphoma

Hodgkin lymphoma and non-Hodgkin lymphoma together are the third most common form of malignancy in children and adolescents [39]. Non-Hodgkin lymphoma is most frequent in children younger than 15 years, whereas Hodgkin lymphoma is predominantly diagnosed in teenagers. Pediatric lymphomas are staged using the Lugano classification for Hodgkin lymphoma [40] and the International Pediatric Non-Hodgkin Lymphoma Staging System for non-Hodgkin lymphoma [41].

**Standard imaging procedure:** In Hodgkin lymphoma, [F-18]2-fluoro-2-deoxyglucose (FDG) positron emission tomography (PET)/CT remains the first-line modality for staging and response assessment, providing both structural and functional metabolic information [40]. In non-Hodgkin lymphoma the current guidelines propose to perform chest radiograph, ultrasonography (US) of the abdomen and cranial/spinal MRI if indicated, except for the B cell non-Hodgkin lymphoma, where FDG PET or whole-body MRI is recommended.

**Review and comments on the literature:** Because both PET and contrast-enhanced CT involve substantial radiation exposure and children often undergo several PET/CT examinations during the course of treatment, there is an increasing interest in the use of whole-body MRI as a good radiation-free alternative. Several studies have shown that whole-body MRI is feasible even in children [2, 8, 29, 42]. Table 1 shows proposed whole-body MRI sequences for use in children with lymphoma.

**Table 1:** Proposed magnetic resonance imaging protocol at 1.5 T in lymphoma (please also refer to Technical Considerations)

**Sequence**	**T1-W TSE**	**T2-W STIR**	**DWI**	**T2-W STIR/SPAIR**^**a**^	**ceT1-W FS**^**a**^
**Orientation**	Coronal	Coronal	Axial	Axial	Axial
**Respiratory motion compensation**	Breath hold (thorax and abdomen)	Respiratory triggering (thorax and abdomen)	Free breathing	Respiratory triggering (thorax and abdomen)	Breath hold^b^
**Anatomical coverage**	Head to groin	Head to groin	Head to groin	Head to groin	Head to groin

*ceT1-W* contrast-enhanced T1-weighted, *DWI* diffusion-weighted imaging, *FS* fat saturation, *SPAIR* spectrally adiabatic inversion recovery, *STIR* short tau inversion recovery, *T1-W* T1-weighted, *T2-W* T2-weighted, *TSE* turbo spin echo

^a^Either axial T2-W STIR/SPAIR or ceT1-W FS, optional

^b^Free breathing with radial imaging

**Staging**: In a study by Punwani et al. [29], the authors reported very good agreement for nodal and extranodal disease involvement between whole-body MRI compared to an FDG PET/CT reference standard, despite only using STIR for whole-body MRI. Because of the clear visualization of lymphoid tissue, diffusion-weighted imaging (DWI) is a potentially interesting additional sequence to use for evaluating lymphoma. Regacini et al. [30] showed an excellent sensitivity for staging pediatric lymphoma compared to contrast-enhanced CT using coronal STIR and DWI sequences. However, other studies could not demonstrate the additional value of DWI to conventional MRI sequences in staging pediatric lymphoma [6, 8]. This could be related to the fact that both benign and malignant nodes demonstrate impeded diffusion. Therefore, the detection of lymph nodes in whole-body DWI is still mainly based on size criteria. Overall, in pediatric Hodgkin lymphoma, whole-body MRI with DWI agreed with an FDG PET/CT-based reference for disease stage in 82–85% of cases [8, 42]. Interestingly, a study performed by Latifoltojar et al. [42] used a highest b value of 500 s/mm^2^. The authors acknowledged that using higher b values could decrease perceptual errors for extranodal disease assessment. Indeed, higher b values are preferred to optimize background body signal suppression and improve lesion conspicuity, in particular in organs/regions with normal intrinsic diffusion restriction.

**Response assessment**: Early recognition of therapy response or failure to chemotherapy enables better selection of children who need more or less intensive therapy. The concept of early response assessment with FDG PET/CT in lymphoma has received considerable attention in the last few years, although it is still not officially recommended outside clinical trials [40]. The role of whole-body MRI in response assessment in pediatric lymphoma is still under investigation. Mayerhoefer et al. [43] showed in 64 adults with lymphoma that whole-body MRI with DWI could serve as a feasible alternative for FDG PET/CT during follow-up and treatment response assessment. Several, mostly pilot, studies compared the quantitative data from FDG PET/CT (standardized uptake value [SUV]) with DWI (apparent diffusion coefficient [ADC] values) for early response assessment, with inconclusive results. They reported presence or absence of an inverse correlation between ADC and SUV [22, 23, 29, 44]. Latifoltojar et al. [42] recently published their results of a prospective study in 55 children with Hodgkin lymphoma and showed that whole-body MRI was correct in 66% and underestimated response in 26% of cases during interim response assessment.

According to the currently available literature, whole-body MRI is a good alternative for contrast-enhanced CT in staging pediatric lymphoma. However, FDG PET with low-dose CT for attenuation correction remains vital for interim response assessment and therefore cannot be omitted during staging or interim response assessment in lymphoma.

**Key recommendation in pediatric lymphoma**: Whole-body MRI is a good alternative for contrast-enhanced CT in staging pediatric lymphoma. However, for FDG-avid lymphomas, 18F-FDG PET remains vital for staging and response assessment.

### Neuroblastoma

Neuroblastoma is a neoplasm arising from primordial neural crest cells and is the second most common solid extracranial tumor in children, accounting for about 6% of all childhood cancers [45]. It can occur anywhere along the sympathetic chain from neck to pelvis, with the adrenal medulla as the most common site at presentation. In about 70% of cases, metastatic disease is observed at diagnosis, including the liver, lymph nodes, bone marrow, cortical bone and skin. About 90% of cases are diagnosed before the age of 6 years. The outcome is variable because neuroblastoma can spontaneously regress in children younger than 1 year, whereas in older children it can cause death despite aggressive treatment with surgery, chemotherapy, bone marrow transplantation and radiotherapy [46].

**Standard imaging procedure:** Accurate staging is pivotal for planning treatment. Furthermore, a systematic assessment of the relationship between the primary tumor and the adjacent structures according to a series of imaging-defined risk factors is mandatory to evaluate tumor resectability [47]. Typically, CT or MRI, iodine-123 metaiodobenzylguanidine (I-123 MIBG) scintigraphy and bone marrow biopsies are used to evaluate local and metastatic disease at diagnosis and during treatment. Further imaging modalities (e.g., FDG PET in MIBG-negative tumors) and frequency of imaging in follow-up are also related to risk groups.

**Review and comments on the literature:** The availability of studies on the role of whole-body MRI in neuroblastoma is still very limited. Furthermore, some of them included children not only with neuroblastoma, but also with other common pediatric tumors. In 2003, Pfluger et al. [48] retrospectively studied 28 people with neuroblastoma who had 50 I-123 MIBG scans in combination with 50 MRI studies. MRI studies included fast spin-echo T1-weighted and T2-weighted images, STIR images and contrast-enhanced T1-weighted images of suspected lesions. They concluded that MRI showed a higher sensitivity and I-123 MIBG a higher specificity, but that integrated imaging with both I-123 MIBG and MRI allowed an increase in both sensitivity and specificity.

In 2013, Siegel et al. [7] compared whole-body MRI with conventional imaging for detecting distant metastases in children with common malignant tumors. Sixty-six children with newly diagnosed lymphoma, neuroblastoma or soft-tissue sarcoma were selected for image review and analysis [7]. The authors concluded that the non-inferior accuracy for diagnosing distant metastases (e.g., pulmonary metastases) was not established for the use of whole-body MRI compared with conventional methods. However, improved accuracy was seen with whole-body MRI in children with non-lymphomatous tumors [7]. In 2014, Kembhavi et al. [49] assessed the diagnostic accuracy of whole-body MRI for metastatic disease in people with small round cell tumors including neuroblastoma, primitive neuroectodermal tumor and rhabdomyosarcoma by comparison with routine staging procedures. Whole-body MRI studies included coronal T1-W and STIR sequences. They concluded that whole-body MRI had high diagnostic accuracy for evaluating metastatic disease to the marrow [49]. On the contrary, the detection rate of nodal metastases was less when whole-body MRI was compared with conventional staging, and chest CT was still essential for accurate evaluation of lung metastases.

Diffusion-weighted imaging can be effectively integrated in MRI studies in children with neuroblastic tumors: Peschmann et al. [50] observed in 19 people that ADC values at diagnosis differed significantly between malignant and benign neuroblastic tumors. Furthermore, low baseline ADC was predictive of tumor progression and relapse. With therapy, increasing ADC appeared to predict relapse-free survival, whereas a decreasing ADC during therapy was an indicator of poor prognosis [50]. Very recently, Ishiguchi et al. [19] studied the role of whole-body DWI with background body suppression using only singular high b value imaging in detection of lymph node and bone metastases from neuroblastoma. Thirteen people underwent both 18F-FDG PET/CT and whole-body DWI with background body suppression. According to the results of this study, whole-body DWI with background body suppression showed a similar level of sensitivity for detecting lymph node metastases to that of FDG PET/CT [19]. However, without ADC mapping, the specificity was poor in this study [19].

The studies on the role of whole-body MRI in neuroblastoma are still scarce, based on a limited number of patients, and in some cases with contradictory results. Nevertheless, whole-body MRI could be a promising, radiation-free modality for detecting bone and bone marrow metastases (Table 2 indicates proposed sequences). Furthermore, the ADC value based on DWI of the primary tumor could be a good indicator of outcome. However, there is still a strong need for prospective large cohort studies to validate the role of whole-body MRI in children with neuroblastoma.

**Table 2:** Proposed magnetic resonance imaging protocol in neuroblastoma (please also refer to Technical Considerations)

**Sequence**	**T2-W STIR**	**T2-W STIR**^**a**^	**DWI**	**T2-W STIR/SPAIR**	**ceT1-W FS**
**Orientation**	Coronal	Sagittal	Axial	Axial	Axial
**Respiratory motion compensation**	Respiratory triggering (thorax and abdomen)	Free breathing	Free breathing	Respiratory triggering (thorax and abdomen)	Breath hold^b^
**Anatomical coverage**	Whole-body	Spine	Whole-body or affected regions	Head to groin	Head to groin

*ceT1-W* contrast-enhanced T1-weighted, *DWI* diffusion-weighted imaging, *FS* fat saturation, *SPAIR* spectrally adiabatic inversion recovery, *STIR* short tau inversion recovery, *T1-W* T1-weighted, *T2-W* T2-weighted, *TSE* turbo spin echo

^a^Optional

^b^Free breathing with radial imaging

**Key recommendations in neuroblastoma**: Whole-body MRI could play a role as an ancillary study for detecting bone and bone marrow disease, while performing MRI for evaluation of the primary tumor. Prospective multicenter studies are needed to validate the role of whole-body MRI versus the current reference standards in pediatric neuroblastoma.

### Sarcomas

Pediatric sarcomas are a heterogeneous group of rare tumors, most of which are highly malignant. They account for approximately 10–15% of solid malignancies in childhood and adolescence. Soft-tissue sarcomas are divided into two groups: rhabdomyosarcomas and non-rhabdomyosarcoma soft-tissue sarcomas. The most common primary malignant bone tumors are osteosarcomas and Ewing sarcomas.

**Standard imaging procedure:** A multidisciplinary diagnostic and therapeutic approach is mandatory in all cases and should be carried out by a reference center [51, 52]. In soft-tissue sarcomas, conventional imaging and staging as recommended by international study groups consist of high-resolution MRI for local disease and loco-regional lymph nodes, CT for lung metastases, and bone scintigraphy for skeletal metastases. Imaging with FDG PET/CT is optional [51, 52] — depending on local availability (European Paediatric Soft-tissue Sarcoma Study Group rhabdomyosarcoma [EpSSG RMS2005] protocol, German Cooperative Weichteilsarkom Studiengruppe [CWS] guidance 2012) — or is recommended at baseline and in cases of suspected tumor recurrence (Children’s Oncology Group Soft Tissue Sarcoma Committee). Bone sarcomas additionally require radiographs of the primary tumor and the search for skip lesions in the same extremity by MRI.

**Review and comments on the literature:** Apart from bone scintigraphy, whole-body imaging was not generally recommended [51, 52] until the most recent EpSSG guideline, which recommends performing FDG PET/CT (MR) for staging at baseline and after three courses of chemotherapy. Its application is especially important in suspected disseminated disease because a curative therapeutic approach requires the local control of each single lesion [52, 53]. The complete depiction of the whole body from head to toe is mandatory, be it to detect all skeletal metastases [26], distant metastases in unexpected localizations [54], or a small primary of a disseminated alveolar rhabdomyosarcoma in a hand or foot [55] (Table 3).

**Table 3:** Proposed magnetic resonance imaging protocol in sarcoma (please also refer to Technical Considerations)

**MR sequence**	**T2-W STIR/SPAIR**	**T2-W STIR/SPAIR**	**DWI**	**ceT1-W FS**	**T2-W STIR HR**^**a**^	**T1-W HR before and with FS after contrast**^**a**^
**Orientation**	Coronal	Axial	Axial	Axial	2 planes, depending on tumor site	Multiple planes^b^
**Respiratory motion compensation**	Respiratory triggering (thorax and abdomen)	Respiratory triggering (thorax and abdomen)	Free breathing	Breath hold^a^	Free breathing	Free breathing
**Anatomical coverage**	Whole body^c^	Head to groin	Whole-body or affected regions	Head to groin	Primary tumor with dedicated coil	Primary tumor with dedicated coil

*ceT1-W* contrast-enhanced T1-weighted, *DWI* diffusion-weighted imaging, *FS* fat saturation, *HR* high resolution, *SPAIR* spectrally adiabatic inversion recovery, *STIR* short tau inversion recovery, *T1-W* T1-weighted, *T2-W* T2-weighted

^a^Optional for dedicated imaging of primary tumor site and single metastases; please refer to EpSSG rhabdomyosarcoma imaging guideline for further recommendations

^b^At least two planes after contrast administration

^c^Especially in alveolar rhabdomyosarcoma, extremities should be completely depicted [55]

Given the young patient age and the high number of follow-up examinations, a modality without exposure to ionizing radiation such as whole-body MRI would be preferable. Whole-body MRI allows for excellent contrast resolution of soft tissue and bone marrow. It provides versatile options such as DWI and contrast enhancement. With a thoroughly planned imaging protocol and a thoughtful choice of MR coils, one examination might simultaneously give an overview of the tumor spread as well as high-resolution images of a tumor site.

Although most experts employ coronal STIR sequences, there is, however, neither a universally accepted standard protocol nor a clearly defined set of assessment criteria. Comparative evaluation of whole-body MRI is therefore difficult. Several studies found that for sarcomas and other solid tumors, whole-body imaging conducted as PET/CT or whole-body MRI outperforms conventional imaging in the detection of metastases [1, 7, 15, 54, 56]. Other results are, however, inconsistent. In older studies, whole-body MRI has been found to be equally sensitive [27] as well as less sensitive [1] as compared to FDG PET. It is also known to detect fewer lung metastases than conventional imaging [7]. PET/CT has, because of its metabolic information, higher sensitivity for detecting nodal disease [7]. An early study showing the possible impact of whole-body MRI on patient outcome was limited by small numbers [5]. A meta-analysis on the detection of skeletal metastases in children with primary solid tumors found data to be too scarce and heterogeneous to recommend whole-body MRI as an alternative. However, 3/5 of these studies included PET as a reference standard, which is also not generally recommended [26]. Qualified studies on the role of diffusion-weighted imaging are also rare, especially in children with rhabdomyosarcoma [57]. DWI might be helpful in staging and therapy monitoring, although diffusion restriction is not specific for malignant lesions and is also typical of normal lymphatic tissue. In osteosarcoma, DWI helps to predict tumor response to neo-adjuvant therapy [58], but larger prospective studies need to be performed.

It seems reasonable that in highly malignant sarcomas the most effective search for metastases and relapses should be preferred even if associated with radiation exposure. However, bone scintigraphy is the only whole-body modality that is mandatory in basic imaging, whereas FDG PET/CT is only optional. To detect relapse, the use of cross-sectional imaging has not been demonstrated to be more beneficial or cost-effective than clinical assessment and chest radiographs alone. Thus, prospective clinical studies are needed, first to define patients who would benefit from whole-body imaging, and second to identify the most effective of the available modalities (whole-body MRI, PET/CT or PET/MRI). Whole-body MRI might, at present, be considered in addition to basic imaging recommendations for children who would probably benefit from whole-body imaging without increasing their cumulative radiation dose.

**Key recommendations in sarcomas**: Whole-body MRI could be considered in addition to imaging recommendations of international oncology groups. Children with sarcoma who have disseminated disease might benefit from whole-body MRI without increasing their cumulative radiation dose.

### Langerhans cell histiocytosis

Langerhans cell histiocytosis is characterized by accumulation of clonal CD1a-positive immature dendritic cells, so-called Langerhans cell histiocytosis cells, together with eosinophils, macrophages, lymphocytes and osteoclast-like giant cells. In children younger than 15 years, incidence of Langerhans cell histiocytosis is 4–5 cases per million per year [59]. Langerhans cell histiocytosis can affect many organs, including the skeleton, skin, lymph nodes, liver, lungs, spleen, hematopoiesis and central nervous system. Previously, Langerhans cell histiocytosis included three entities: eosinophilic granuloma, Hand–Schüller–Christian disease and Letterer–Siwe disease, but at present it is classified as unifocal, single-system multifocal, or multifocal multi-system disease [59]. Children with single-system or organ Langerhans cell histiocytosis, such as skeleton, skin or lymph nodes, have an excellent prognosis and need minimal or sometimes no treatment at all. The outcome of children with multisystem Langerhans cell histiocytosis can range from spontaneous resolution to fatal outcome despite treatment. Therefore, it is of the utmost importance to stratify these patients according to unifocal versus multifocal disease.

**Standard imaging procedure:** Diagnostic evaluation and treatment are based on the ongoing LCH-IV International Collaborative Treatment Protocol for Children and Adolescents with Langerhans cell histiocytosis (EudraCT Nr.: 2011–001699-20). According to this protocol, diagnostic imaging at onset should include an abdominal US study for evaluating size and structure of the liver and spleen, a chest radiograph and a radiologic skeletal survey. Functional imaging like bone scan or FDG PET is optional and can be performed in addition to the skeletal survey. Chest CT is needed in case of lung involvement, whereas head CT or MRI should be performed in case of craniofacial lesions or mastoid involvement. Neurologic abnormalities or suspected endocrine abnormalities require a head MRI, and MRI of the spine is necessary in cases of suspected vertebral lesions.

**Review and comments on the literature:** Langerhans cell histiocytosis can be a multisystem and multifocal disease. Therefore, whole-body MRI could be an excellent method for evaluating the whole body in one examination. Nevertheless, very few studies with preliminary results on the role of whole-body MRI in Langerhans cell histiocytosis are available. The number of children included in these studies is limited (range 2 to 46) [3, 25, 60–62]. Three of five available studies investigated the role of whole-body MRI at diagnosis for primary staging or follow-up; in two studies only the role of whole-body MRI at diagnosis was investigated [3, 25, 60–62]. STIR sequence was performed in all studies; in one of them it was the only sequence performed [61]. T1-W fast spin-echo (FSE) sequences with and without contrast enhancement were performed in two studies [60, 62], T1-W FSE without contrast enhancement in one study [25], and T1-W FSE with just contrast enhancement in another study [3]. All studies included coronal and sagittal images, whereas only one study also included axial images [62].

The standard of reference for comparison with whole-body MRI was skeletal survey or plain radiographs in four studies [3, 25, 60, 61], in combination with skeletal scintigraphy in three of them [3, 60, 61]. Histopathology or follow-up imaging was the standard of reference in another study [62]; this study compared the performances of FDG PET with whole-body MRI in Langerhans cell histiocytosis. Sensitivity and specificity of whole-body MRI for lesion detection in Langerhans cell histiocytosis (81% and 47%, respectively) were reported only in the study by Mueller et al. [62], whereas Kim et al. [60] reported a sensitivity of 99%.

According to the results of their study, Steinborn et al. [25] concluded that whole-body MRI had a higher detection rate of bony lesions than a skeletal survey, and they therefore suggested that whole-body MRI be the imaging modality of choice for assessing skeletal involvement in Langerhans cell histiocytosis. Similarly, Goo et al. [3] and Laffan et al. [61] reported that whole-body MRI can be an excellent imaging tool for assessing skeletal involvement. Furthermore, Goo et al. [3] concluded that whole-body MRI can also detect extraskeletal disease, and they suggested that changes in signal intensity on STIR and T1-W contrast-enhanced images could demonstrate response to treatment in active lesions. Mueller et al. [62] studied the diagnostic value of FDG PET and MRI in pediatric Langerhans cell histiocytosis. They concluded that MRI showed higher sensitivity than FDG PET in lesion detection, whereas FDG PET showed higher specificity. Interestingly, they also reported that FDG PET was more accurate than MRI in evaluating disease activity after chemotherapy because they observed persisting residual contrast enhancement and T2 hyperintensity in lesions with no residual FDG uptake on PET. Probably the most interesting results — based on the largest case series so far — come from a recent study by Kim et al. [60]. They concluded that whole-body MRI had higher detectability for Langerhans cell histiocytosis lesions than skeletal survey and bone scintigraphy, with no significant differences in the number of false-positives per patient, while the three modalities had comparable accuracy in the initial staging. Table 4 presents a summary of these studies’ findings. Table 5 presents a whole-body MRI protocol for Langerhans cell histiocytosis.

**Table 4:** Summary of published data on whole-body magnetic resonance imaging in Langerhans cell histiocytosis

	**Goo et al.** [3]	**Laffan et al.** [61]	**Steinborn et al.** [25]	**Mueller et al.** [62]	**Kim et al.** [60]
**Number of patients**	9	2	14	15	46
**Staging at diagnosis**	Yes	Yes	Yes	Yes	Yes
**Follow-up**	Yes	No	Yes	Yes	No
**Total number of lesions observed**	NA	NA	NA	53	105
**Number of lesions observed on primary staging**	NA	NA	NA	25	105
**Number of lesions observed on follow-up**	NA	NA	NA	28	NA
**Sequences**	STIR, T1-W FSE CE	STIR	T1-W FSE, STIR	T1-W FSE, STIR, T1-W FSE CE + dedicated study of the brain with T1-W FSE, T2-W FSE, FLAIR, T1-W FSE CE	STIR, T1-W FSE, T1-W FSE CE
**Acquisition planes**	Coronal, sagittal limited to the trunk	Coronal and sagittal	Coronal and sagittal	Axial, coronal, sagittal	Coronal, sagittal
**Standard of reference**	RX skeletal survey, bone scintigraphy	Skeletal scintigraphy and/or plain radiographs	RX skeletal survey	Histopathology and/or follow-up of lesions. Whole-body MRI performances were also compared with 18F-FDG PET	Histopathology and/or follow-up of lesions
**Sensitivity**	NA	NA	NA	81%	99%
**Specificity**	NA	NA	NA	47%	NA

*CE* contrast-enhanced, *DWI* diffusion-weighted imaging, *FDG PET* [F-18]2-fluoro-2-deoxyglucose positron emission tomography, *FLAIR* fluid-attenuated inversion recovery, *FS* fat saturation, *FSE* fast spin echo, *NA* not available, *RX* radiograph, *SPAIR* spectrally adiabatic inversion recovery, *STIR* short tau inversion recovery, *T1-W* T1-weighted, *T2-W* T2-weighted, *TSE* turbo spin echo

**Table 5:** Proposed whole-body magnetic resonance imaging protocol in Langerhans cell histiocytosis (please also refer to Technical Considerations)

**Sequences**	**T1-W TSE**	**T2-W STIR**	**T2-W STIR**	**T2-W STIR**^**a**^	**T1-W TSE**^**a**^
**Orientation**	Coronal	Coronal	Sagittal	Axial	Axial
**Respiratory motion compensation**	Breath hold (thorax and abdomen)	Respiratory triggering (thorax and abdomen)	Free breathing	Free breathing	Free breathing
**Anatomical coverage**	Head to toe	Head to toe	Spine	Head	Head

*STIR* short tau inversion recovery, *T1-W* T1-weighted, *T2-W* T2-weighted, *TSE* turbo spin echo

^a^Optional

According to the scientific literature available, whole-body MRI could be the imaging modality of choice for assessing skeletal involvement at onset, thus replacing radiologic skeletal survey and bone scintigraphy. Conventional radiologic studies could be performed only of bones with positive findings on whole-body MRI. Whole-body MRI should include at least T1-W FSE and STIR images in coronal and sagittal planes, whereas it is unclear whether T1-W contrast-enhanced images improve the accuracy of the study. None of the published studies assessed the role of diffusion-weighted imaging. The role of whole-body MRI during follow-up seems to be questionable because persisting signal abnormalities could be caused by post-therapy tissue reorganization. In addition, the role of whole-body MRI in evaluating extraskeletal disease is promising but not finally approved. New and larger prospective multicenter studies are therefore needed.

**Key recommendations in Langerhans cell histiocytosis**: Whole-body MRI could replace skeletal survey and bone scintigraphy for assessing skeletal involvement at onset. Its role in assessment of extraosseous disease and follow-up is still unclear. Thanks to its high sensitivity and accuracy, whole-body MRI should always be considered in children with Langerhans cell histiocytosis at onset, especially if sedation is not needed.

## Screening in cancer predisposition disorders

Cancer predisposition syndromes comprise entities characterized by a risk of development of specific tumors or co-occurrence of tumors caused by a germ-line mutation in one or more cancer-related genes [63]. These can manifest from infancy to adulthood and are of unknown penetrance, variable incidence, and differing age of onset. At least 10% of children with cancer harbor a disease-associated pathogenic variant in a known cancer predisposition gene. Common cancer predisposition syndromes in children include Li–Fraumeni syndrome, constitutional mismatch repair deficiency syndrome (CMMRD), hereditary paraganglioma, pheochromocytoma syndrome, rhabdoid tumor predisposition syndrome, hereditary retinoblastoma, and Neurofibromatosis 1. Cancers typically related to Li–Fraumeni syndrome are osteosarcoma, adrenocortical carcinoma, brain tumors, soft-tissue sarcoma and premenopausal breast cancer [37, 64]. Lower-risk sites are the bowel, bone marrow and skin. Children with hereditary retinoblastoma are at risk of developing osteosarcoma; soft-tissue sarcoma; nasal/orbital tumor; melanoma; and lung, bladder, breast and uterine carcinoma [65]. Cancers related to CMMRD are brain tumors, gastrointestinal and hematologic malignancies [31]. Identification of an underlying cancer predisposition syndrome could lead to a recommendation of health surveillance or prophylactic surgery.

**Standard imaging procedure:** The role of imaging in cancer predisposition syndromes is to be part of tumor surveillance and to allow for detection of tumor recurrence. Furthermore, imaging enables assessment of individuals and family members identified as being at risk for tumors on the basis of an abnormal genetic test result. Because of the increased sensitivity of children to ionizing radiation, most surveillance imaging protocols rely more on US and MRI (whole-body MRI) than nuclear medicine and CT [10, 11]. Because the cancer spectrum in cancer predisposition syndromes is age-dependent, screening modalities and frequency change depending on the gender and age of the patient (e.g., adrenocortical cancer risk in very young children and high breast cancer risk in young woman with Li–Fraumeni syndrome; specific surveillance of the gastrointestinal tract, the central nervous system, and the hematopoietic system in infancy; and surveillance of the genitourinary tract in older children with CMMRD).

**Review and comments on the literature:** Variations in screening protocols arise from institutional preference and technological capabilities. Whole-body MRI can be performed with the additional use of regional MRI (e.g., dedicated brain MRI in Li–Fraumeni syndrome, CMMRD and hereditary retinoblastoma, or targeted MRI of specific organs and extremities) [10, 11]. It provides head-to-toe coverage, displayed mostly as whole-body fused coronal images. A coronal fluid-sensitive 2-D sequence (STIR) represents the key sequence of a whole-body MRI screening protocol. T1-weighted images are applied for better tissue characterization (in particular regarding bone marrow signal). Whole–body DWI, typically acquired axially, is now incorporated into whole-body pediatric protocols. However, DWI and ADC measurements at diagnosis and follow-up must still be validated [66]. Generally, whole-body MRI can be performed without general anesthesia in children with a developmental age of 6 years and older.

In a retrospective study of 50 whole-body MRIs in 24 pediatric patients with cancer predisposition syndromes, Anupindi et al. [31] demonstrated that whole-body MRI is a method with a very high negative predictive value (NPV; 100%). Friedman et al. [38] reported an NPV of 97% in a total of 41 whole-body MRI screening tests performed in survivors of hereditary retinoblastoma. This would indicate that children with a normal whole-body MRI are unlikely to have a tumor. Villani et al. [65] reported in 2016 that long-term compliance with a comprehensive surveillance protocol in individuals with pathogenic *TP53* variants is associated with improved long-term survival. Their protocol included physical examination and frequent biochemical and imaging studies (consisting of whole-body MRI, brain MRI, breast MRI, mammography, abdominal and pelvic ultrasound, and colonoscopy). O’Neill et al. [34] recently postulated that it is feasible to use whole-body MRI for tumor surveillance in pediatric patients with Li–Fraumeni syndrome in his group of 22 patients. In Neurofibromatosis 1, routine MRI surveillance is not recommended unless children are symptomatic or with an already diagnosed tumor [37].

From these studies, it can be concluded that whole-body MRI complemented with targeted imaging (organ, brain) when indicated can properly fulfill the need for a radiation-free method for early tumor detection in pediatric cancer predisposition syndromes. However, Anupindi et al. [31] showed that most people in their study (96%) had incidental findings of no significant clinical impact. False-positive findings can be a potential risk in whole-body MRI surveillance of cancer predisposition syndromes. Therefore, the benefits of screening with whole-body MRI must be weighed against risks, including potential technique-related issues, false-positive imaging findings and costs. Last but not least, whole-body MRI recommendations for tumor surveillance usually do not include the age group of children ages 4-6 months and 6 years.

**Summary of screening in cancer predisposition syndromes (Table **6**):** Whole-body MRI can reveal multifocal disease in cancer predisposition syndromes and can be complemented by dedicated regional studies with small field-of-view (FOV) imaging if indicated. The absence of ionizing radiation is an advantage in the pediatric population. Standard whole-body MRI methodology and imaging protocols for screening of many pediatric cancer predisposition syndromes are not clearly established. While modernization of MRI devices is in progress globally, the same technical level of MRI scanners, technologists’ expertise and radiologists’ experience in cancer predisposition syndromes interpretation cannot be guaranteed yet. There is a need for large prospective multicenter studies to establish the role, effectiveness and costs of screening with whole-body MRI in pediatric cancer predisposition syndromes. Despite the lack of standardization in the screening and evaluation of multifocal lesions in pediatric patients, whole-body MRI is certainly among the imaging methods of first choice.

**Table 6:** Whole-body magnetic resonance imaging surveillance in cancer predisposition syndromes

**Disease**	Body sequences				**MRI brain, dedicated**
	**T2-W STIR coronal**	**T1-W TSE coronal**	**DWI axial (coronal reconstruction)**	**T2-W SPAIR axial**^**a**^**(thorax, abdomen, breath hold or respiratory triggering)**	
Li–Fraumeni syndrome	Whole-body MRI every 12 months from diagnosis^b^				Every 6 months^b^
Congenital mismatch repair syndrome	Whole-body MRI every 12 months from 6 years to 8 years				Not indicated
Hereditary retinoblastoma	Whole-body MRI every 12 months from 8 years to 10 years				Every 6 months to 5 years of age
Neurofibromatosis 1 and neurofibromatosis 2	Whole-body MRI baseline scan at 16–20 years (no need for whole-body MRI surveillance unless symptomatic or detected tumor); whole-body MRI baseline				Indicated

*DWI* diffusion-weighted imaging, *SPAIR* spectrally adiabatic inversion recovery, *STIR* short tau inversion recovery, *T1-W* T1-weighted, *T2-W* T2-weighted, *TSE* turbo spin echo

^a^Optional

^b^Whole-body MRI and MRI brain interleaved at 6-month intervals if no general anesthetic is needed

**Key recommendations in cancer predisposition syndromes:** Whole-body MRI plays an increasing role in the surveillance of children with cancer predisposition syndromes. In cancer predisposition syndrome surveillance with whole-body MRI, knowledge of the tumor spectrum per syndrome and knowledge of the imaging characteristics of specific tumors and normal variants are mandatory to improve lesion detection and reduce false-positive findings.

## General technical considerations

Given a larger field of view and a more stable magnetic field, a 1.5-tesla (T) scanner might be preferable to a 3.0-T device for whole-body MRI. The diagnostic performance of MRI depends substantially on the sequence type and scan parameters, especially the spatial resolution. The variety of technical options renders it a versatile modality, but for the same reasons it is highly operator-dependent and difficult to standardize. The often long duration of the examination directly affects patient comfort. Thus, a modular approach consisting of basic and advanced sequences is useful [4, 13, 17, 18, 67, 68]. An example of a whole-body MRI protocol from the pediatric radiology department at the University Hospital Tübingen is shown in Table 7. The modular concept facilitates the adjustment of the protocol to a particular indication and also improves the reproducibility and comparability of individual examinations.

**Table 7:** Example of a modular concept of whole-body magnetic resonance imaging protocol

**Module**	**Region**	**Sequence**	**Orientation**/**phase encoding direction**	**Base matrix (FOV adapted to child’s size)**	**Remarks**
**Basic module**	Whole body	2-D STIR TSE/FSE	Coronal/FH	384	
	Head and neck	2-D STIR TSE/FSE	Axial/AP	384	Head to aortic arch
	Thorax	2-D T2-W TSE/FSE fat-saturated navigator	Axial/AP	384	Radial k-space sampling with respiratory triggering
	Abdomen and pelvis	2-D T2-W TSE/FSE fat-saturated navigator	Axial/AP	384	Radial k-space sampling with respiratory triggering
**DWI**	Whole body	2-D EPI (SPAIR) 2 b values: 0 and 1,000 s/mm^2^	Axial/AP	128	Coronal MPR; ADC map; calculation of high b value ≥1,200 s/mm^2^
**CE**	Whole body	3-D T1-W GRE (VIBE/Dixon)	Axial/AP	288–320	If possible breath hold; coronal MPR
**Optional local imaging: brain, abdominal organs, spine, extremities**					

*ADC* apparent diffusion coefficient, *AP* anterior-to-posterior, *CE* contrast-enhanced, *DWI* diffusion-weighted imaging, *EPI* echoplanar imaging, *FH* feet-to-head, *FSE* fast spin echo, *GRE* gradient echo, *MPR* multiplanar reconstruction, *SPAIR* spectral attenuated inversion recovery, *STIR* short tau inversion recovery, *TSE* turbo spin echo, *VIBE* volumetric interpolated breath-hold examination

In young children the company of a parent, adequate introduction to the examination, and distraction by audio or video equipment during the scan help to reduce motion artifacts and even the necessity of sedation or general anesthesia. These preparations might seem time-consuming, but their influence on image quality should not be underestimated.

In agreement with previous publications, we propose a high-resolution fat-saturated T2-weighted sequence (e.g., STIR) with slice thicknesses of 3–4 mm in coronal orientation as the mainstay of the basic module. To avoid limitations due to partial volume effects, additional sections in transverse orientation in the regions of the head, neck and trunk are recommended [4, 13, 17, 18, 67–69]. For reduction of motion artifacts, radial k-space acquisition and navigator techniques are helpful. Electrocardiography-triggered sequences might improve the depiction of small lesions in the mediastinum or in the lung. Sequences or reconstructions in sagittal orientation are necessary when focusing on spinal pathologies. Advanced modules comprise (whole-body) diffusion-weighted and T1-weighted sequences before and after administration of contrast medium. After contrast injection, fat suppression is mandatory.

In addition to whole-body examinations, dedicated imaging of body regions such as orbits, hands and feet, and the evaluation of small lesions in residual or relapsing disease require optimized equipment and coil configuration [4, 13, 17, 18, 67–69]. Particularly in the case of children who can only be examined under general anesthesia or sedation, additional imaging (e.g., local MRI) might be replaced by this comprehensive approach. This advantage often outweighs the necessary extension of the examination time [16].

In the literature, most protocol recommendations for whole-body MRI in children and adolescents give a fixed FoV, resolution matrix and slice thickness and thus a fixed voxel size. However, as is typical in pediatric MRI, these sequence parameters should be adapted to the indication as well as the various body sizes from infant to adolescent because the spatial resolution is significant for diagnostic sensitivity [70]. Especially in cases where sequences are acquired in a single (coronal) orientation, a sufficiently high resolution is crucial. Therefore, the following approach might be helpful: based on a given resolution matrix (e.g., 256 × 256 - 384 × 384), the voxel size is automatically reduced by adjusting the FOV to the child’s size and by moderately adjusting the slice thickness to a smaller body size, resulting in relatively constant anatomical resolution/image information. Therefore, for morphological assessment we recommend a voxel size in children younger than 1 year of 1x1x3 mm^3^ in the coronal plane and of 0.8×0.8×3.0 mm^3^ in the transverse plane. However, the subsequent signal loss should be compensated (e.g., by increase of the in-phase oversampling or the signal averaging), resulting in a longer acquisition time. Therefore, an optimal compromise between tolerable signal-to-noise ratio loss and measurement time should be found. It is advantageous that the number of blocks/stations in the Z-direction and thus the total acquisition time can be reduced based on the body geometry of small children. The use of predefined protocols optimized to different body sizes facilitates adaptation in daily practice.
